# Carry on caring: infected females maintain their parental care despite high mortality

**DOI:** 10.1093/beheco/arab028

**Published:** 2021-04-21

**Authors:** Tom Ratz, Katy M Monteith, Pedro F Vale, Per T Smiseth

**Affiliations:** Institute of Evolutionary Biology, School of Biological Sciences, University of Edinburgh, Charlotte Auerbach Road, Edinburgh EH9 3FL, UK; Institute of Evolutionary Biology, School of Biological Sciences, University of Edinburgh, Charlotte Auerbach Road, Edinburgh EH9 3FL, UK; Institute of Evolutionary Biology, School of Biological Sciences, University of Edinburgh, Charlotte Auerbach Road, Edinburgh EH9 3FL, UK; Institute of Evolutionary Biology, School of Biological Sciences, University of Edinburgh, Charlotte Auerbach Road, Edinburgh EH9 3FL, UK

**Keywords:** antimicrobial peptide expression, immunity, parental care, *Nicrophorus vespilloides*, reproductive investment

## Abstract

Parental care is a key component of an organism’s reproductive strategy that is thought to trade-off with allocation toward immunity. Yet, it is unclear how caring parents respond to pathogens: do infected parents reduce care as a sickness behavior or simply from being ill or do they prioritize their offspring by maintaining high levels of care? To address this issue, we investigated the consequences of infection by the pathogen *Serratia marcescens* on mortality, time spent providing care, reproductive output, and expression of immune genes of female parents in the burying beetle *Nicrophorus vespilloides*. We compared untreated control females with infected females that were inoculated with live bacteria, immune-challenged females that were inoculated with heat-killed bacteria, and injured females that were injected with buffer. We found that infected and immune-challenged females changed their immune gene expression and that infected females suffered increased mortality. Nevertheless, infected and immune-challenged females maintained their normal level of care and reproductive output. There was thus no evidence that infection led to either a decrease or an increase in parental care or reproductive output. Our results show that parental care, which is generally highly flexible, can remain remarkably robust and consistent despite the elevated mortality caused by infection by pathogens. Overall, these findings suggest that infected females maintain a high level of parental care, a strategy that may ensure that offspring receive the necessary amount of care but that might be detrimental to the parents’ own survival or that may even facilitate disease transmission to offspring.

## Teaser

Sick parents face the dilemma of whether to prioritize their own health and reduce care to their offspring or prioritize their offspring by maintaining high levels of care. We investigated this by infecting breeding females of burying beetles with a pathogen and found that infected females maintained normal levels of care despite suffering from increased mortality. Parents might thus seek to prioritize care to the offspring over their own health.

## INTRODUCTION

When infected by a pathogen, animals often alter their behaviors and social interactions (Hart 1988; Kelley et al. 2003; Adelman and Martin 2009; Vale et al. 2018). This change in behavior may occur as a side effect of lethargy (Adelman and Martin 2009) or it may represent what is known as sickness behavior; a strategic decision to shift resources toward immune defense by reducing activity levels (Van Kerckhove et al. 2013; Lopes 2014) and costly social interactions (Bos et al. 2012). Lethargy may be a consequence of the pathogen negatively impacting on the host’s ability to remain active, thus leading to reduced mobility (e.g., Cameron et al. 1993; Bradley and Altizer 2005), foraging (e.g., Levri and Lively 1996; Venesky et al. 2009), and social activity (Lopes et al. 2016). Lethargy may also be associated with sickness behavior, an adaptive adjustment to fight the infection that allows the host to diverge resources from nonessential activities, such as social interactions, to the immune system (Hart 1988; Exton 1997; Johnson 2002). When individuals interact with family members, sickness behavior may also help reduce the risk of disease transmission to close kin (Heinze and Walter 2010; Stroeymeyt et al. 2018) as a possible kin-selected behavior (Shakhar and Shakhar 2015; Shakhar 2019). However, recent empirical evidence shows that sick individuals often maintain their social interactions with close kin (Lopes et al. 2018; Stockmaier et al. 2020). Yet, empirical studies testing the effects of infection on social behavior toward close kin are still scarce, with most studies being based on immune challenges (injecting with heat-killed pathogens or products from pathogens; e.g., Aubert et al. 1997; Bonneaud et al. 2003; Stockmaier et al. 2020) that exclude potential effects of the pathogen on host’s behavior.

Parental care is a key component of an organism’s reproductive strategy in many birds, mammals, and insects (Royle et al. 2012) that is thought to trade-off with allocation of resources toward immunity (Richner et al. 1995). Caring parents incur costs of care in terms of increased energy expenditure, reduced opportunities for additional reproductive attempts, reduced survival, and/or reduced future reproductive success (Williams 1966). Parental care enhances offspring growth and/or survival by neutralizing environmental hazards to offspring, including risks associated with starvation, predation, parasitism, and competition (Royle et al. 2012). Thus, when infected by a pathogen, parents face the dilemma of whether to shift allocation toward immunity at the expense of maintaining their level of parental care or maintain the level of parental care at the expense of increasing their allocation toward immunity. Parents that reduce their level of care to increase their immune response would risk impairing their offspring’s growth and survival, whereas parents that maintain their level of care would risk falling ill by not mounting an adequate immune response. Experimental studies using immune challenges found that female laboratory mice tend to maintain their level of care and maintain normal offspring growth and survival (Aubert et al. 1997), while house sparrows drastically reduce their food provisioning at the cost of reduced offspring survival (Bonneaud et al. 2003). Thus, it is unclear how caring parents balance allocation toward parental care and immunity in response to infection: do infected parents reduce or maintain their level of care, and is there a trade-off between the magnitude of the immune responses and the level of parental care?

Here, we investigated how parents balance their allocation toward parental care and immunity in response to infection in the burying beetle *Nicrophorus vespilloides*. This is an ideal system to investigate this issue because it is one of the few insects with extensive parental care. Parental care includes provisioning of food to larvae, defense against predators and infanticidal conspecific intruders, and production of antimicrobials that enhances the offspring’s growth and survival (Eggert et al. 1998; Scott 1998; Smiseth et al. 2003; Rozen et al. 2008). Burying beetles show changes in immunity during parental care (Steiger et al. 2011), which include differential expression of antimicrobial peptides (Jacobs et al. 2016; Ziadie et al. 2019). Parents may mount a personal immune response that helps them deal with pathogens. However, there is also evidence that parents invest in social immunity that benefits the offspring but is costly to the parents (Cotter and Kilner 2010b; Ziadie et al. 2019). Social immunity in burying beetles occurs as parents coat the carcass used for breeding with exudates with potent antibacterial activity (Cotter and Kilner 2010b), which reduces microbial load and improves the offspring’s survival (Rozen et al. 2008).

To test for a causal effect of infection on parental care and immunity, we monitored the amount of care provided by infected females that were inoculated with live bacteria, immune-challenged females that were inoculated with heat-killed bacteria, injured females that were injected with buffer, and untreated control females. We also monitored their lifespan and overall reproductive output. In parallel, we quantified the personal and social immune responses of females in each treatment by measuring the expression of genes encoding antimicrobial peptides, namely *attacin-4*, *cecropin-1*, *coleoptericin-1* (personal immunity; Jacobs et al. 2016), and *PGRP-SC2* (social immunity; Parker et al. 2015; Ziadie et al. 2019). If females respond to infection by shifting their allocation toward immunity, we would expect infected and/or immune-challenged females to show a reduction in parental care and an increase in the overall expression of immune genes. Alternatively, if females respond to infection and/or immune challenges by shifting allocation toward current reproduction, we would predict infected and/or immune-challenged females to maintain their level of parental care and show a reduction in the overall expression of immune genes. Assuming there is a trade-off between personal and social immunity (Cotter and Kilner 2010a), we expect an increase in the expression of genes involved in personal immunity relative to the expression of genes involved in social immunity if infected and/or immune-challenged females shift allocation toward their own immunity (Cotter et al. 2013). Alternatively, we would expect a reduction in the expression of genes involved in personal immunity relative to the expression of genes involved in social immunity if infected and/or immune-challenged females shift allocation toward current reproduction.

## MATERIALS AND METHODS

### Origin and rearing of experimental beetles

Experimental beetles originated from wild individuals collected in the Hermitage of Braid and Blackford Hill Local Nature Reserve, Edinburgh, UK. The beetles had been maintained in a large outbred population (200–300 individuals were bred per generation) under laboratory conditions for at least five generations before the start of our experiment. Nonbreeding adult beetles were housed in individual transparent plastic containers (12 × 8 × 2 cm) filled with moist soil under constant temperature at 20 °C, 16:8 h light:dark photoperiod, and ad libitum access to organic beef as food supply.

### Experimental design and procedures

To investigate the effects of infection on parental care, reproductive output, and immunity, we used a group of untreated control females (*N*_Control_ = 61) and three groups of experimental females: infected females that were inoculated with the pathogenic bacteria *Serratia marcescens* (*N*_Infected_ = 58), immune-challenged females that were inoculated with heat-killed *S. marcescens* (*N*_Challenged_ = 70), and injured females that were injected with buffer (*N*_Injured_ = 56). At the beginning of the experiment, each individual virgin female was randomly assigned an unrelated male partner and transferred to a larger plastic container (17 × 12 × 6 cm) lined with moist soil and containing a freshly thawed mouse carcass of a standardized size (19.97–23.68 g; Livefoods Direct, Sheffield). We weighed each female on the day before the anticipated hatching date (i.e., 2 days after the onset of egg-laying; Smiseth et al. 2006). We then placed females in an individual plastic vial plugged with cotton. Females remained in this vial until we applied the treatment (see details below), after which they were transferred into a new large container containing fresh soil and supplied with their original carcass. We left the eggs to develop in the old container, while males were discarded. We separated the females from the eggs so that we could allocate each female with an experimental brood of 15 same-aged larvae of mixed maternal origin. We removed the male to avoid any potential effects of male parental care buffering against effects of the experimental treatment on the female. Male removal has no effect on the developing brood under laboratory conditions (Smiseth et al. 2005). We next set up experimental broods of 15 larvae by collecting newly hatched larvae emerging in the soil, starting the day following the separation of females and eggs. A brood size of 15 larvae is within the range of brood sizes on the range of carcass sizes used in our experiment (10–40 larvae on 19–24-g carcasses; Smiseth and Moore 2002). We generated experimental broods by pooling larvae that had hatched from eggs laid by multiple females (Smiseth et al. 2007). We used a standardized brood size that was comprised of 15 larvae of a known age to avoid any potential confounding effects of variation in the number and age of the larvae on maternal behavior (Smiseth et al. 2003; Ratz and Smiseth 2018). Given that parents will kill any larvae that emerge on the carcass before their own eggs have hatched (Müller and Eggert 1990), we only allocated an experimental brood to a female once her own eggs had hatched.

### Bacterial preparation

We chose *S. marcescens* (strain DB11) as an appropriate natural bacterial pathogen for *Nicrophorus vespillodies*. *Serratia marcescens* is a gram-negative bacterium commonly found in the soil and on decomposing carrion (El Sanousi et al. 1987; Hejazi and Falkiner 1997). It has been shown to infect several insect species and is known to cause mortality in both eggs and larva of *N. vespilloides* (Jacobs et al. 2014; Wang and Rozen 2018). Pilot tests confirmed that *S. marcescens* increased female mortality (Ratz et al., unpublished data) but only when injected above a certain concentration and volume (see below). We also note that our pilot tests showed that stabbing with *Pectobacterium carotovorum* and *Pseudomonas aeruginosa* and injections with *Pseudomonas entomophila* had no detectable effect on female mortality.

To grow the *S. marcescens* culture, we inoculated 10 mL of Luria-Bertani (LB) broth (Fisher Scientific) with 200 μL of a frozen 25% glycerol suspension from a single isolated *S. marcescens* colony. The culture was aerobically incubated overnight in an orbital shaker at 140 rpm and 30 °C. On the day of infection, the overnight culture was diluted 1:10 into fresh LB broth and incubated under the same conditions until the culture had reached the mid-log growth phase (OD_600_ 0.6–0.8). Optical density was checked using a microplate absorbance reader at an absorbance of 600 nm. The mid-log phase culture was pelleted by centrifugation (15 min, 4 °C, 2500 rpm) and the supernatant removed. The pellet was then resuspended in sterile phosphate buffer saline (PBS, pH 7.4) and adjusted to OD_600_ 1. The final inoculum OD_600_ was calculated as described in Siva-Jothy et al. (2018). The final inoculum was split into two tubes; one tube was heated to 70 °C for 45 min killing the bacteria and allowing for an immune-challenged treatment group, while the other tube was kept as a live culture for the infected treatment group.

### Infection procedure

On the day preceding the expected date of hatching, we randomly allocated each female to an experimental treatment group. Females from all treatment groups were first anesthetized by releasing CO_2_ into their individual tube for 40 s. Control females were then returned to their vials to recover for 30 min, while experimental females were placed on a CO_2_ pad under a dissecting microscope. Injured females were wounded using a glass needle attached to a microinjector (Nanoject II, Drummond Scientific Co.) to inject 0.552 µL of sterile PBS buffer. This allowed us to simulate an injury without causing infection. We used the same protocol to inject immune-challenged females with 0.552 µL of heat-killed *S. marcescens* solution and infected females with 0.552 µL of OD_600_ 1 live *S. marcescens* solution (~1.3 million colony-forming units). We performed the injection by introducing the needle through the soft cuticle that joins the thorax and the abdomen on the ventral side (Reavey et al. 2014). Once injected, experimental females were returned to their vials to recover for 30 min. Following recovery, we next moved control and injected females back to the large containers containing their carcasses.

### Maternal care, female weight change, female mortality, and offspring performance

We recorded the amount of care provided by each female 24 h (±15 min) after we placed the larvae on the carcass, which corresponded to 48 h (±4 h) after females were handled and/or injected. This enabled us to monitor female behavior at a point in time that females would be expected to mount a potential immune response, which is generally expected to start within the first day and continue during several days following a bacterial challenge (e.g., Korner and Schmid-Hempel 2004; Haine et al. 2008). We performed direct observations under red light for 30 min, recording maternal behavior every 1 min in accordance with established protocols (e.g., Smiseth and Moore 2002, 2004; Ratz and Smiseth 2018). We recorded maternal care as food provisioning, defined as when there was mouth-to-mouth contact between the female and at least one larva, and carcass maintenance, defined as when the female was excavating the soil around the carcass or coating the carcass with antimicrobial secretions. We conducted the behavioral observations blindly with respect to treatment as it was not possible for the observer to identify the experimental treatments.

Females and their broods were then left undisturbed until larvae completed their development, at which stage they left the mouse carcass to disperse into the soil. At dispersal, we weighed the female, counted the number of larvae, and weighed the brood. We estimated weight gain over the reproductive attempts by the female as the difference in body mass between egg-laying and larval dispersal. We estimated larval survival as the difference between the final brood size at dispersal and the initial brood size at hatching (i.e., 15 larvae) and mean larval mass as the total brood mass divided by brood size.

### Hemolymph sampling, RNA extraction, reverse transcription, and qPCR

To examine the effects of the treatment on the female’s immune response, we quantified the expression of genes coding for antimicrobial peptides (AMPs) by quantitative real-time polymerase chain reaction (qRT-PCR). We focused on the expression of the four following genes: *attacin-4*, *cecropin-1*, *coleoptericin-1,* and *PGRP-SC2*. We focused on these genes because they are known to have a role in the immune response against gram-negative bacteria, such as *S. marcescens* (Imler and Bulet 2005; Vilcinskas, Mukherjee, et al. 2013; Vilcinskas, Stoecker, Schmidtberg, et al. 2013) and there is some knowledge about their function in personal or social immunity in this species (Parker et al. 2015; Jacobs et al. 2016; Ziadie et al. 2019): *attacin-4*, *cecropin-1*, and *coleoptericin-1* seem to play a role mainly in personal immunity (Jacobs et al. 2016), while *PGRP-SC2* plays a role in social immunity (Parker et al. 2015; Ziadie et al. 2019).

In parallel with the behavioral observation, we randomly selected a subset of females for RNA extraction, which included 13 control, 14 injured, 17 immune-challenged, and 14 infected females. We removed each of these females from their containers 48 h (±4 h) after infection and placed them in an individual plastic vial plugged with cotton. We then anesthetized each female with CO_2_ as described above. Once anesthetized, we extracted hemolymph from each female placed on a CO_2_ pad by puncturing the soft cuticle behind the thorax with a micropine and then drawing hemolymph with a 10-μL glass capillary. We sampled 2–10 μL of hemolymph per female and transferred it into 1.5-μL microtubes containing 100 μL of TRIzol reagent (Invitrogen, Life Technologies). All hemolymph samples were then stored at −70 °C until RNA extraction.

RNA extractions were performed using the standard phenol-chloroform method and included a DNase treatment (Ambion, Life Technologies). The RNA purity of eluted samples was confirmed using a Nanodrop 1000 Spectrophotometer (version 3.8.1). cDNA was synthesized from 2 μL of the eluted RNA using M-MLV reverse transcriptase (Promega) and random hexamer primers and then diluted 1:1 in nuclease free water. We performed quantitative RT-PCR on an Applied Biosystems StepOnePlus machine using Fast SYBR Green Master Mix (Applied Biosystems). We used a 10-μL reaction containing 1.5 μL of 1:1 diluted cDNA, 5 μL of Fast SYBR Green Master Mix, and 3.5 μL of a primer stock containing both forward and reverse primers at 1 μM suspended in nuclease free water (final reaction concentration of each primer 0.35 μM). For each cDNA sample, two technical replicates were performed for each set of primers and the average threshold cycle (Ct) was used for analysis.

Primers were designed based on amino acid sequences provided on Kyoto Encyclopedia of Genes and Genomics (KEGG) or supplementary information provided by Jacobs et al. (2016; KEGG: *PGRP-SC2*, *rlp7*; Jacobs et al. 2016: *attacin-4*, *coleoptericin-1*, and *cecropin-1*). Briefly, the amino acid sequence was entered into the Basic Local Alignment Search Tool (BLAST) on NCBI.gov, the accession number producing the most similar alignments within *N. vespilloidies* was selected, and the corresponding nucleotide sequence used for primer design in Primer3 (version 4.1.0) and Beacon Designer (Premier Biosoft International). All primers were obtained from Sigma-Aldrich Ltd; Attacin-4_Forward: 5’ GCATTTACACGCACAGACCT 3’, Attacin-4_Reverse 5’ CGGCAACTTTACTTCCTCCG 3’; Cecropin-1_Forward 5’ CGAGCACACAACAGTTCCTT 3’, Cecropin-1_Reverse 5’ ATCAAAGCTGCGATGACCAC 3’; Coleoptericin-1_Forward 5’ GAAACGGTGGTGAACAGGTG 3’, Coleoptericin-1_Reverse 5’ GAGTCTTGGGGAACGGGAA 3’; PGRP-SC2_Forward 5’ CGAAGGTCAAGGTTGGGGTA 3’, PGRP-SC2_Reverse 5’ GTTCCGATGACACAGATGCC 3’. We used *rpl7* as an endogenous reference gene, following Jacobs et al. (2014, 2016); Rpl7_Forward 5’ GTCGGCAAGAACTTCAAGCA 3’, Rpl7_Reverse 5’ TCCCTGTTACCGAAGTCACC 3’. For each pair of primers, the annealing temperature (T_a_) was optimized and the efficiency (Eff) of each primer pair calculated by 10-fold serial dilution of a target template (each dilution was assayed in duplicate): attacin-4: T_a_ = 59 °C, Eff = 102.21%; cecropin-1: T_a_ = 59.5 °C, Eff = 102.26%; coleoptericin-1 T_a_ = 61.6°C, Eff = 101.86%; PGRP-SC2: T_a_ = 60.2 °C, Eff = 99.84%; Rpl7: T_a_ = 60 °C Eff = 98.25%.

### Statistical analysis

All statistical analyses were conducted using R version 3.6.0 (R Development Core Team 2011) loaded with the packages *car* (Fox et al. 2016), *MASS* (Ripley et al. 2017), and *glmmTMB* (Brooks et al. 2017). We analyzed data on parental care using a zero-inflated binomial model. We used analysis of variance (ANOVA) models to analyze normally distributed data; that is, female weight change over breeding and mean larval mass at dispersal. We used a quasi-Poisson model to analyze data on female life span and a binomial model to analyze data on larval survival. Note that we did not use a Cox proportional hazards model to analyze female survival as this was not necessary given that we had data on life span of all females, allowing us to compare the life spans of females in the different treatment groups and because our data did not satisfy the assumption of proportional hazards (Therneau 2015; χ  ^2^ = 12.0, *P* = 0.007). All models included the treatment as a fixed effect with four levels (i.e., infected, immune-challenged, injured, and control females). To account for potential effects of brood size on maternal care (Smiseth et al. 2003; Ratz and Smiseth 2018), we also included brood size at the time of observation as covariate in the model analyzing maternal care. We ran pairwise comparisons using a Tukey’s test with the Bonferroni correction whenever the treatment had a significant effect.

To analyze data on gene expression, we first calculated the expression of a gene of interest relative to the reference gene *rpl7* to obtain ΔC_T_ values (Livak and Schmittgen 2001). We then used ANOVA models to test for the effects of the experimental treatment on the ΔC_T_ values of each gene. Whenever the treatment had a significant effect on gene expression, we ran pairwise comparisons using a Tukey’s test with the Bonferroni correction.

Among the 245 females, we sacrificed a subset of 59 females to sample hemolymph, of which one was excluded because not enough hemolymph was obtained. Among the remaining females, we excluded 55 additional females from our analysis on maternal care, life span, and larval survival because their eggs failed to hatch (*N* = 10), there were not enough larvae to allocate them a brood (*N* = 25), the female or the whole brood died before the observation (*N* = 12), no behavioral data were collected (*N* = 1), or the heat-kill treatment failed (*N* = 7). The final sample of the behavioral and life-history data included 33 control females, 32 injured females, 33 immune-challenged females, and 33 infected females. Likewise, we excluded 9 broods (control females: *N* = 4; injured females: *N* = 3; immune-challenged females: *N* = 2) from our analysis on mean larval mass at dispersal because no larvae survived to dispersal.

## RESULTS

**Figure 1:**
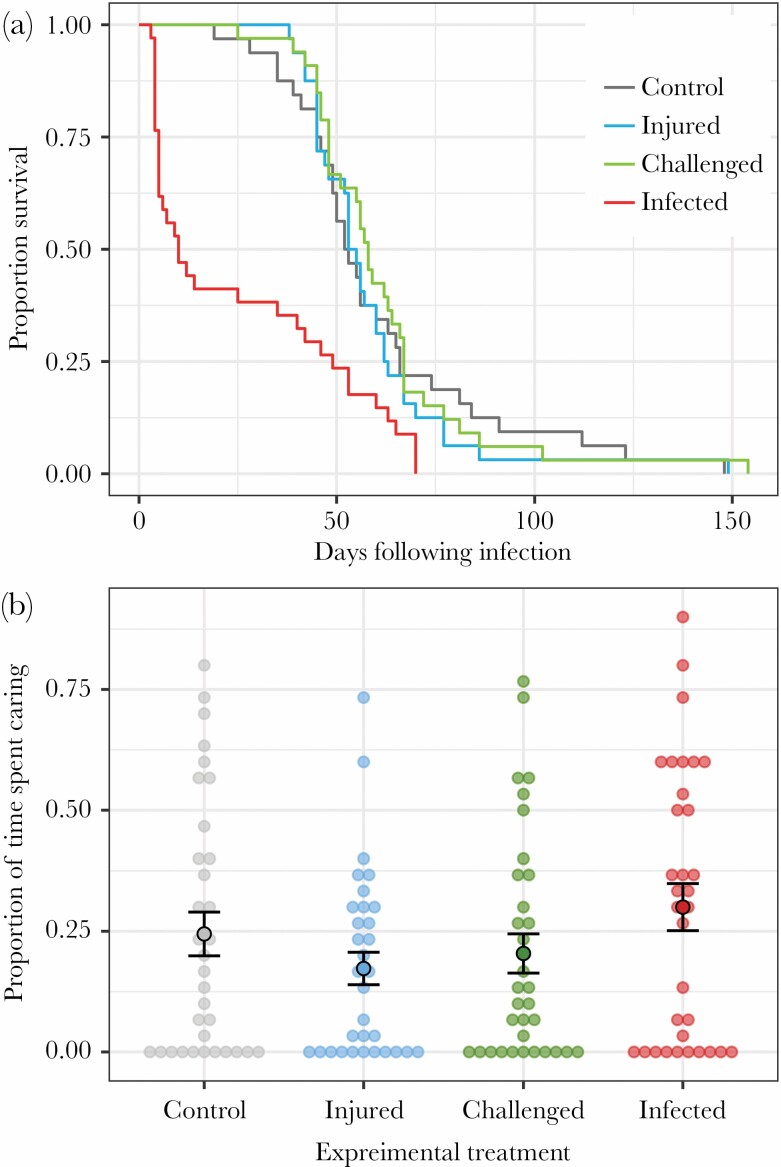
Proportion of females alive over time after the day the treatment was applied (a). Effects of the experimental treatment on maternal care (b). Open circles represent individual data, closed circles and bars represent means ± standard errors.

There was a significant effect of treatment on female life span (Figure 1a; χ  ^2^ = 52.1, degrees of freedom [df] = 3, *P* < 0.001), which reflected that infected females had an average life span that was 75% shorter than females from any other treatment group (Table 1). There was no significant effect of treatment on the amount of care provided by females (Figure 1b; χ  ^2^ = 6.63, df = 3, *P* = 0.085), showing that females maintained a similar level of care to control females regardless of whether they were infected, immune challenged, or injured. There was no effect of brood size at the time of observation on maternal care (χ  ^2^ = 2.62, df = 1, *P* = 0.105). There was no effect of treatment on mean larval mass at dispersal (*F*_3,118_ = 0.613, *P* = 0.608) or survival of the larvae until dispersal (χ  ^2^ = 5.66, df = 3, *P* = 0.129), suggesting that infected, immune-challenged, or injured females maintained a similar level of reproductive output to control females. There was no difference in weight change between females in the different treatments (*F*_3,112_ = 1.42, *P* = 0.239).

**Table 1:** Pairwise comparisons between treatments for the postinfection life span. *P* values were obtained using Tukey’s HSD (honestly significant difference) test and adjusted using the Bonferroni correction

	Postinfection life span			
	Estimate	SE	*Z*	*P*
Injured–control	−0.035	0.117	−0.299	0.991
Challenged–control	0.014	0.115	0.123	0.999
Infected–control	−0.866	0.148	−5.83	**<0.001**
Injured–challenged	−0.049	0.116	−0.424	0.974
Infected–injured	−0.831	0.149	−5.57	**<0.001**
Infected–challenged	−0.880	0.147	−5.97	**<0.001**

Statistically significant *P* values (<0.05) are shown in boldface.

SE standard error.

**Figure 2:**
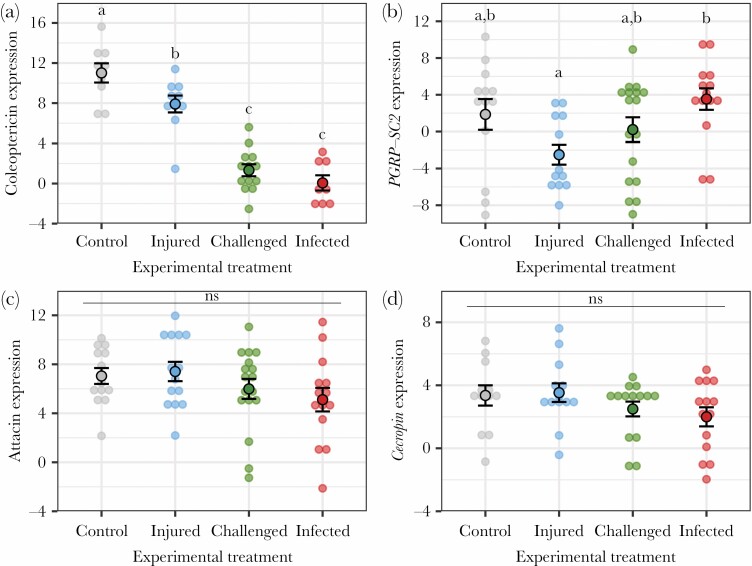
Effects of the experimental treatment on the expression of coleoptericin-1 (a), PGRP-SC2 (b), attacin-4 (c), and cecropin-1 (d). Open circles represent individual data; closed circles and bars represent means ± standard errors. Different letters indicate significant difference from pairwise analyses when *P* < 0.05 and ns indicates no significant difference.

We next investigated the effects of the experimental treatments on the expression of four immune genes. Treatment had a significant effect on the expression of *coleoptericin-1* (Figure 2a; *F*_3,36_ = 42.9, *P* < 0.0001). The expression of this gene was lower in injured females than in control females (Table 2), lower in immune-challenged females than in injured females (Table 2), and similar in immune-challenged and infected females (Table 2). Treatment also had a significant effect on the expression of *PGRP-SC2* (Figure 2b; *F*_3,53_ = 3.47, *P* = 0.022). The expression of this gene was reduced in injured females compared with infected ones (Table 2), while there was no difference in expression between females in any of the other treatment groups (Table 2). We found no significant effect of treatment on the expression of *attacin-4* (Figure 2c; *F*_3,54_ = 1.55, *P* = 0.211) or *cecropin-1* (Figure 2d; *F*_3,50_ = 1.57, *P* = 0.206).

**Table 2:** Pairwise comparisons between treatments for the level of gene expression for *coleoptericin-1* and *PGRP-SC2*. *P* values were obtained using Tukey’s HSD test and adjusted using the Bonferroni correction

	*coleoptericin-1*				*PGRP-SC2*			
	Estimate	SE	*T*	*P*	Estimate	SE	*t*	*P*
Injured–control	−3.10	1.13	−2.74	**0.045**	−4.36	1.98	−2.19	0.136
Challenged–control	−9.68	1.06	−9.07	**<0.001**	−1.62	1.86	−0.886	0.811
Infected–control	−10.9	1.19	−9.15	**<0.001**	1.66	1.94	0.856	0.826
Injured–challenged	6.58	1.03	6.36	**<0.001**	−2.71	1.86	−1.45	0.471
Infected–injured	−7.84	1.16	−6.72	**<0.001**	6.03	1.94	3.09	**0.016**
Infected–challenged	−1.26	1.10	−1.14	0.666	3.32	1.82	1.81	0.275

Statistically significant *P* values (<0.05) are shown in boldface.

SE standard error.

## DISCUSSION

Here, we show that infected and immune-challenged females altered their expression of immune genes and that infected females had a shortened life span compared to other females. Despite the heightened mortality of infected females, we found no evidence for a difference between infected, immune-challenged, injured, and control females in their level of care or their reproductive output. Altogether, our findings indicate that infected females maintained their level of care despite changing their immune gene expression and clear evidence that the pathogen shortened their life span. This strategy may allow infected females to provide the necessary amount of care to ensure the growth and survival of their offspring but could be detrimental to the parents by increasing their mortality and may potentially even facilitate disease transmission to offspring. Below, we discuss the broader implications of these findings to our understanding of the effects of infection on parental behavior and social interactions between caring parents and their dependent offspring.

As expected, we found that infected females altered their expression of immune genes and had a considerably shortened life span, confirming that infection with *S. marcescens* had the intended effect of triggering an immune response and making infected females sick. Immune-challenged females showed a similar change in the expression of immune genes as infected females but suffered no corresponding reduction in their life span. Thus, our results confirm that the shortened life span of infected females was caused by the pathogen rather than being a byproduct of the immune response. Taken together, our results confirm that *S. marcescens* is a potent pathogen in *N. vespilloides*. These results are similar to previous studies in *N. vespilloides* reporting elevated mortality as a result of an infection by *Photorhabdus luminescens* (Miller and Cotter 2017, 2018), but contrast with other studies documenting no change in mortality following inoculation with other bacteria (Reavey et al. 2015; Ratz et al., unpublished data). This difficulty in establishing experimental infections in this species may reflect that it breeds on decomposing carcasses, which means they regularly will be in close contact with potential pathogens (Jacobs et al. 2014; Wang and Rozen 2018). Our study species might thus be resistant to a wide variety of bacterial strains, such as *Bacillus subtilis* (Reavey et al. 2015), *P. carotovorum*, *P. aeruginosa*, *P. entomophila*, or *S. marcescens* at low doses and concentrations (Ratz et al., unpublished data) that are pathogenic in many other insect species. Our results show that, as long as *S. marcescens* is injected in relatively high dose and concentration, it can successfully establish an infection in *N. vespilloides*, activate the immune system, and greatly increase mortality.

Our main finding was that infected females maintained their level of care and their reproductive output, despite showing changes in immune gene expression and suffering negative fitness consequences of infection as indicated by their shortened life span. Although a comparison between breeding and nonbreeding females is needed to determine whether females prioritize parental care over their own immunity, the maintenance of high levels care by infected females may impede their allocation of resources toward immunity. By maintaining their level of care, infected females may ensure that offspring receive the necessary amount of care and produce offspring with a similar survival and body size as offspring of uninfected females. This strategy might allow infected females to maintain their reproductive output (e.g., Arundell et al. 2014) but might come at a cost in terms of reduced survival and future reproductive success. Burying beetles can produce multiple broods (Creighton et al. 2009) and tend to gain mass during first reproduction, which is positively correlated with life span (Gray et al. 2018). Infection should reduce fitness given that infected females are likely to die before producing an additional brood. This is because approximately 60% of infected females in our study had died by 17 days after infection (compared with 0% of control females; Figure 1a), which corresponds to the minimum duration necessary for 1) the current brood to complete larval development (about 7 days; Smiseth et al. 2003, 2005), 2) the female to find and secure a new carcass (which are rare; Scott 1998), and 3) the female to produce eggs and care for the new brood (which would take another 10 days; Ford and Smiseth 2017). An alternative explanation for our results is that infected females perceived their chance of survival and future reproduction to be low and that they, therefore, maintained a high level of care as a terminal investment response (Williams 1966) as suggested by prior studies on *N. vespilloides* reporting high reproductive output following an immune challenge (e.g., Cotter et al. 2010; Reavey et al. 2014, 2015; Farchmin et al. 2020). Yet, we found no evidence for an increase in reproductive investment in immune-challenged or infected females, as would be expected under terminal investment. Thus, rather than mounting a terminal investment response, we suggest that infected females maintained their level of care to provide the necessary amount of care to ensure offspring growth and survival, potentially at a cost to females in terms of reduced survival.

Our finding that infected females maintained their level of care also shows that infections do not necessarily induce sickness behavior. Infected hosts often show reduced social interactions (Hart 1988; Kelley et al. 2003; Vale et al. 2018), which may be the result of lethargy (i.e., reduced activity levels) of the host associated with sickness (Adelman and Martin 2009), the host actively avoiding costly social interactions (Lopes et al. 2016; Sah et al. 2018), uninfected individuals avoiding an infected host (Curtis 2014), or the pathogen manipulating the host’s behavior (Moore 2002; Hughes et al. 2012). Yet, this reduction in social behavior is not always observed, depending on the social context (Lopes et al. 2012; Adamo et al. 2015), and parents that are sick might maintain their level of care and interactions with offspring (Stockmaier et al. 2020). Because parental care and parent–offspring interactions can have a large impact on the reproductive output of organisms, we propose that infected parents might prioritize their allocation in reproduction by maintaining necessary care and social interactions with their offspring. In species with biparental care, infected females might be able to reduce their level of care (and thereby increase their immune response) without harming their offspring if the male parent compensate for the reduction in female care. If so, male compensation could temper the negative effect of infection on female life span. Thus, we encourage future studies to compare the responses of infected females in the contexts of biparental care and uniparental care.

Our last finding was that females from the different treatment groups showed different levels of expression in two immune genes (i.e., *coleoptericin-1* and *PGRP-SC2*), while there was no difference in the expression of other immune genes (i.e., *attacin-4* and *cecropin-1*). The expression of *coleoptericin-1*, a gene involved in personal immunity (Parker et al. 2015; Jacobs et al. 2016), was lower in immune-challenged and infected females than in injured and control females. This was opposite to our prediction and surprising given prior evidence showing that immune-challenged and infected females upregulate personal immunity genes, such as *defensin* (Ziadie et al. 2019), in response to immune challenges (Reavey et al. 2014). In contrast, the expression of *PGRP-SC2*, a social immunity gene, as it provides offspring with antimicrobial protection (Parker et al. 2015; Ziadie et al. 2019), was higher in infected females than in injured females. Given that there was no difference in immune gene expression between immune-challenged and infected females, it seems unlikely that the pathogen suppressed the immune system in our study species. Instead, these results might reflect immune responses to the presence of a pathogen or, in the case of immune-challenged females, to the presence of cues from a potential pathogen. Thus, our finding that infected females had lower personal immunity and maintained normal levels of social immunity points toward a shift in investment toward current reproduction. This suggests that infected and immune-challenged females maintained their investment in social immunity that benefits larval survival, which would support the idea that infected females overall sought to maintain their allocation toward current reproduction.

Our findings have important implications for our understanding of parental behavior under the risk of infection by showing that infected females maintained a high level of care despite the fact that infections could expose their offspring to the pathogen. Thus, our results show that the level of care is remarkably stable in response to infection, notwithstanding evidence that parents often show a great amount of plasticity in response to other environmental factors, such as resource abundance and the presence of competitors and infanticidal conspecifics (Smiseth and Moore 2002; Hopwood et al. 2015; Georgiou Shippi et al. 2018). Furthermore, behavioral plasticity represents the first mechanism of immunity (Kiesecker et al. 1999; Schaller 2006; Schaller and Park 2011) and might allow infected individuals to reduce the risk of transmission to close kin, including offspring (Shakhar and Shakhar 2015; Shakhar 2019). Our study found no evidence that females transmitted the pathogen to their offspring given that we found no indication that larvae of infected females had higher mortality than larvae of other females. Nevertheless, we urge future studies to consider the potential consequences of disease transmission by caring parents to their offspring (Chakarov et al. 2015). For example, infected parents might be expected to maintain their level of care in situations where the risk of females passing on the pathogen to their offspring is low. In contrast, infected parents might reduce their level of care in situations where the risk of females passing on the pathogen to their offspring is high and where the offspring are not completely dependent on their parents.

In summary, our study shows that infected females maintained their level of parental care and reproductive output despite showing changes in immune gene expression and suffering from greater mortality. Our results demonstrate that parental care, which is generally highly flexible, can remain robust and stable in response to pathogenic infections. The results also suggest that infected females maintain their current reproductive success over survival, which could ensure that offspring receive the necessary amount of care. Our findings stress the need for more studies on infection in species where parents care for and interact with their offspring, as parental care is a fundamental social interaction in all birds and mammals, as well as some amphibians, fishes, and arthropods, and as it can have contradicting effects by buffering against environmental hazards on the one hand and providing a potential route for disease transmission on the other hand.

We thank the City of Edinburgh Natural Heritage Service for permission to collect beetles in their reserve at the Hermitage of Braid and Blackford Hill Local Nature Reserve. We also thank Jon Richardson for assistance with maintaining the laboratory population, Arun Prakash, Sarah Reece, Saudamini Venkatesan, Ferghal Waldron, and Michelle Ziadie for suggestions and useful advice on the experimental set up, and Eevi Savola for helpful discussions regarding the analysis of gene expression data. We are grateful to Allen Moore, Sheena Cotter and two anonymous reviewers for constructive suggestions on an earlier version of this paper. T.R. was supported by the Darwin Trust of Edinburgh.

## Data availability

Analyses reported in this article can be reproduced using the data provided by Ratz et al. (2021).

## References

[CIT0001] Adamo SA, Gomez-Juliano A, LeDue EE, Little SN, Sullivan K. 2015. Effect of immune challenge on aggressive behaviour: how to fight two battles at once. Anim Behav. 105:153–161.

[CIT0002] Adelman JS, Martin LB. 2009. Vertebrate sickness behaviors: adaptive and integrated neuroendocrine immune responses. Integr Comp Biol. 49:202–214. 21665814 10.1093/icb/icp028

[CIT0003] Arundell KL, Wedell N, Dunn AM. 2014. The impact of predation risk and of parasitic infection on parental care in brooding crustaceans. Anim Behav. 96:97–105.

[CIT0004] Aubert A, Goodall G, Dantzer R, Gheusi G. 1997. Differential effects of lipopolysaccharide on pup retrieving and nest building in lactating mice. Brain Behav Immun. 11:107–118. 9299060 10.1006/brbi.1997.0485

[CIT0005] Bonneaud C, Mazuc J, Gonzalez G, Haussy C, Chastel O, Faivre B, Sorci G. 2003. Assessing the cost of mounting an immune response. Am Nat. 161:367–379. 12703483 10.1086/346134

[CIT0006] Bos N, Lefèvre T, Jensen AB, d’Ettorre P. 2012. Sick ants become unsociable. J Evol Biol. 25:342–351. 22122288 10.1111/j.1420-9101.2011.02425.x

[CIT0007] Bradley CA, Altizer S. 2005. Parasites hinder monarch butterfly flight: implications for disease spread in migratory hosts. Ecol Lett. 8:290–300.

[CIT0008] Brooks ME, Kristensen K, van Benthem KJ, Magnusson A, Berg CW, Nielsen A, Skaug HJ, Maechler M, Bolker B. 2017. glmmTMB balances speed and flexibility among packages for zero-inflated generalized linear mixed modeling. R J. 9:378–400.

[CIT0009] Cameron PG, Semlitsch RD, Bernasconi MV. 1993. Effects of body size and parasite infection on the locomotory performance of juvenile toads, *Bufo bufo*. Oikos 66:129–136.

[CIT0010] Chakarov N, Linke B, Boerner M, Goesmann A, Krüger O, Hoffman JI. 2015. Apparent vector-mediated parent-to-offspring transmission in an avian malaria-like parasite. Mol Ecol. 24:1355–1363. 25688585 10.1111/mec.13115

[CIT0011] Cotter SC, Kilner R. 2010a. Personal immunity versus social immunity. Behav Ecol. 21:663–668.

[CIT0012] Cotter SC, Kilner R. 2010b. Sexual division of antibacterial resource defence in breeding burying beetles, *Nicrophorus vespilloides*. J Anim Ecol. 79:35–43. 19627394 10.1111/j.1365-2656.2009.01593.x

[CIT0013] Cotter SC, Littlefair JE, Grantham PJ, Kilner RM. 2013. A direct physiological trade-off between personal and social immunity. J Anim Ecol. 82:846–853. 23363060 10.1111/1365-2656.12047

[CIT0014] Cotter SC, Ward RJ, Kilner RM. 2010. Age-specific reproductive investment in female burying beetles: independent effects of state and risk of death. Funct Ecol. 25:652–660.

[CIT0015] Creighton JC, Heflin ND, Belk MC. 2009. Cost of reproduction, resource quality, and terminal investment in a burying beetle. Am Nat. 174:673–684. 19775240 10.1086/605963

[CIT0016] Curtis VA . 2014. Infection-avoidance behaviour in humans and other animals. Trends Immunol. 35:457–464. 25256957 10.1016/j.it.2014.08.006

[CIT0017] Eggert Ak, Reinking M, Muller JK. 1998. Parental care improves offspring survival and growth in burying beetles. Anim Behav. 55:97–107. 9480676 10.1006/anbe.1997.0588

[CIT0018] El Sanousi SM, El Sarag MSA, Mohamed SE. 1987. Properties of *Serratia marcescens* isolated from diseased honeybee (*Apis mellifera*) larvae. Microbiology 133:215–219.

[CIT0019] Exton MS . 1997. Infection-induced anorexia: active host defence strategy. Appetite. 29:369–383. 9468766 10.1006/appe.1997.0116

[CIT0020] Farchmin PA, Eggert AK, Duffield KR, Sakaluk SK. 2020. Dynamic terminal investment in male burying beetles. Anim Behav. 163:1–7.

[CIT0021] Ford LE, Smiseth PT. 2017. Asynchronous hatching in a nonavian species: a test of the hurry-up hypothesis. Behav Ecol. 28:899–907.

[CIT0022] Fox J, Weisberg S, Adler D, Bates D, Baud-bovy G, Ellison S, Firth D, Friendly M, Gorjanc G, Graves S, et al. 2016. Package “car.” Available from: http://cran.r-project.org/web/packages/car/car.pdf.

[CIT0023] Georgiou Shippi AG, Paquet M, Smiseth PT. 2018. Sex differences in parental defence against conspecific intruders in the burying beetle *Nicrophorus vespilloides*. Anim Behav. 136:21–29.

[CIT0024] Gray FE, Richardson J, Ratz T, Smiseth PT. 2018. No evidence for parent–offspring competition in the burying beetle *Nicrophorus vespilloides*. Behav Ecol. 29:1142–1149.

[CIT0025] Haine ER, Pollitt LC, Moret Y, Siva-Jothy MT, Rolff J. 2008. Temporal patterns in immune responses to a range of microbial insults (*Tenebrio molitor*). J Insect Physiol. 54:1090–1097. 18513740 10.1016/j.jinsphys.2008.04.013

[CIT0026] Hart BL . 1988. Biological basis of the behavior of sick animals. Neurosci Biobehav Rev. 12:123–137. 3050629 10.1016/s0149-7634(88)80004-6

[CIT0027] Heinze J, Walter B. 2010. Moribund ants leave their nests to die in social isolation. Curr Biol. 20:249–252. 20116243 10.1016/j.cub.2009.12.031

[CIT0028] Hejazi A, Falkiner FR. 1997. Serratia marcescens. J Med Microbiol. 46:903–912. 9368530 10.1099/00222615-46-11-903

[CIT0029] Hopwood PE, Moore AJ, Tregenza T, Royle NJ. 2015. Male burying beetles extend, not reduce, parental care duration when reproductive competition is high. J Evol Biol. 28:1394–1402. 26033457 10.1111/jeb.12664

[CIT0030] Hughes DP, Brodeur J, Thomas F. 2012. Host manipulation by parasites. Oxford: Oxford University Press.

[CIT0031] Imler JL, Bulet P. 2005. Antimicrobial peptides in Drosophila: structures, activities and gene regulation. In: Kabelitz D, Schroder JM, editors. Mechanisms of epithelial defense. Vol. 86. Basel, Switzerland: Karger Publishers. p. 1– 21. 10.1159/000086648 15976485

[CIT0032] Jacobs CG, Steiger S, Heckel DG, Wielsch N, Vilcinskas A, Vogel H. 2016. Sex, offspring and carcass determine antimicrobial peptide expression in the burying beetle. Sci Rep. 6:25409. 27139635 10.1038/srep25409 PMC4853764

[CIT0033] Jacobs CG, Wang Y, Vogel H, Vilcinskas A, van der Zee M, Rozen DE. 2014. Egg survival is reduced by grave-soil microbes in the carrion beetle, *Nicrophorus vespilloides*. BMC Evol Biol. 14:208. 25260512 10.1186/s12862-014-0208-x PMC4189599

[CIT0034] Johnson RW . 2002. The concept of sickness behavior: a brief chronological account of four key discoveries. Vet Immunol Immunopathol. 87:443–450. 12072271 10.1016/s0165-2427(02)00069-7

[CIT0035] Kelley KW, Bluthé RM, Dantzer R, Zhou JH, Shen WH, Johnson RW, Broussard SR. 2003. Cytokine-induced sickness behavior. Brain Behav Immun. 17(Suppl 1):S112–S118. 12615196 10.1016/s0889-1591(02)00077-6

[CIT0036] Kiesecker JM, Skelly DK, Beard KH, Preisser E. 1999. Behavioral reduction of infection risk. Proc Natl Acad Sci USA. 96:9165–9168. 10430913 10.1073/pnas.96.16.9165 PMC17750

[CIT0037] Korner P, Schmid-Hempel P. 2004. In vivo dynamics of an immune response in the bumble bee *Bombus terrestris*. J Invertebr Pathol. 87:59–66. 15491600 10.1016/j.jip.2004.07.004

[CIT0038] Levri EP, Lively CM. 1996. The effects of size, reproductive condition, and parasitism on foraging behaviour in a freshwater snail, *Potamopyrus antipodarum*. Anim Behav. 51:891–901.

[CIT0039] Livak KJ, Schmittgen TD. 2001. Analysis of relative gene expression data using real-time quantitative PCR and the 2(-Delta Delta C(T)) Method. Methods. 25:402–408. 11846609 10.1006/meth.2001.1262

[CIT0040] Lopes PC . 2014. When is it socially acceptable to feel sick? Proc Biol Sci. 281:20140218. 24943375 10.1098/rspb.2014.0218 PMC4083780

[CIT0041] Lopes PC, Adelman J, Wingfield JC, Bentley GE. 2012. Social context modulates sickness behavior. Behav Ecol Sociobiol. 66:1421–1428.

[CIT0042] Lopes PC, Block P, König B. 2016. Infection-induced behavioural changes reduce connectivity and the potential for disease spread in wild mice contact networks. Sci Rep. 6:31790. 27548906 10.1038/srep31790 PMC4993150

[CIT0043] Lopes PC, Block P, Pontiggia A, Lindholm AK, König B. 2018. No evidence for kin protection in the expression of sickness behaviors in house mice. Sci Rep. 8:16682. 30420741 10.1038/s41598-018-35174-0 PMC6232183

[CIT0044] Miller CV, Cotter SC. 2017. Pathogen and immune dynamics during maturation are explained by Bateman’s Principle. Ecol Entomol. 42:28–38.

[CIT0045] Miller CVL, Cotter SC. 2018. Resistance and tolerance: the role of nutrients on pathogen dynamics and infection outcomes in an insect host. J Anim Ecol. 87:500–510. 28975615 10.1111/1365-2656.12763

[CIT0046] Moore J . 2002. Parasites and the behavior of animals. Oxford: Oxford University Press.

[CIT0047] Müller JK, Eggert AK. 1990. Time-dependent shifts between infanticidal and parental behavior in female burying beetles a mechanism of indirect mother-offspring recognition. Behav Ecol Sociobiol. 27:11–16.

[CIT0048] Parker DJ, Cunningham CB, Walling CA, Stamper CE, Head ML, Roy-Zokan EM, McKinney EC, Ritchie MG, Moore AJ. 2015. Transcriptomes of parents identify parenting strategies and sexual conflict in a subsocial beetle. Nat Commun. 6:8449. 26416581 10.1038/ncomms9449 PMC4598741

[CIT0049] R Development Core Team . 2011. R: a language and environment for statistical computing. Vienna (Austria): R Foundation for Statistical Computing.

[CIT0050] Ratz T, Monteith KM, Vale PF, Smiseth PT. 2021. Data from: carry on caring: infected females maintain their parental care despite suffering high mortality. Behav Ecol. doi:10.5061/dryad.dfn2z3510. PMC8842341 35169391

[CIT0051] Ratz T, Smiseth PT. 2018. Flexible parents: joint effects of handicapping and brood size manipulation on female parental care in *Nicrophorus vespilloides*. J Evol Biol. 31:646–656. 29468774 10.1111/jeb.13254

[CIT0052] Reavey CE, Silva FW, Cotter SC. 2015. Bacterial infection increases reproductive investment in burying beetles. Insects. 6:926–942. 26529021 10.3390/insects6040926 PMC4693179

[CIT0053] Reavey CE, Warnock ND, Vogel H, Cotter SC. 2014. Trade-offs between personal immunity and reproduction in the burying beetle, *Nicrophorus vespilloides*. Behav Ecol. 25:415–423.

[CIT0054] Richner H, Christe P, Oppliger A. 1995. Paternal investment affects prevalence of malaria. Proc Natl Acad Sci USA. 92:1192–1194. 7862659 10.1073/pnas.92.4.1192 PMC42664

[CIT0055] Ripley B, Venables B, Bates DM, Hornik K, Gebhardt A, Firth D. 2017. Package “MASS”. Available from: http://cran.r-project.org/web/packages/MASS/MASS.pdf.

[CIT0056] Royle NJ, Smiseth PT, Kölliker M. 2012. The evolution of parental care. Oxford: Oxford University Press.

[CIT0057] Rozen DE, Engelmoer DJ, Smiseth PT. 2008. Antimicrobial strategies in burying beetles breeding on carrion. Proc Natl Acad Sci USA. 105:17890–17895. 19001269 10.1073/pnas.0805403105 PMC2584725

[CIT0058] Sah P, Mann J, Bansal S. 2018. Disease implications of animal social network structure: a synthesis across social systems. J Anim Ecol. 87:546–558. 29247466 10.1111/1365-2656.12786

[CIT0059] Sarkar A, Harty S, Johnson KV, Moeller AH, Archie EA, Schell LD, Carmody RN, Clutton-Brock TH, Dunbar RIM, Burnet PWJ. 2020. Microbial transmission in animal social networks and the social microbiome. Nat Ecol Evol. 4:1020–1035. 32572221 10.1038/s41559-020-1220-8

[CIT0060] Schaller M . 2006. Parasites, behavioral defenses, and the social psychological mechanisms through which cultures are evoked. Psychol Inq. 17:96–101.

[CIT0061] Schaller M, Park JH. 2011. The behavioral immune system (and why it matters). Curr Dir Psychol Sci. 20:99–103.

[CIT0062] Scott MP . 1998. The ecology and behavior of burying beetles. Annu Rev Entomol. 43:595–618. 15012399 10.1146/annurev.ento.43.1.595

[CIT0063] Shakhar K . 2019. The inclusive behavioral immune system. Front Psychol. 10:1004. 31130904 10.3389/fpsyg.2019.01004 PMC6509541

[CIT0064] Shakhar K, Shakhar G. 2015. Why do we feel sick when infected—can altruism play a role? PLoS Biol. 13:e1002276. 26474156 10.1371/journal.pbio.1002276 PMC4608734

[CIT0065] Siva-Jothy JA, Prakash A, Vasanthakrishnan RB, Monteith KM, Vale PF. 2018. Oral bacterial infection and shedding in *Drosophila melanogaster*. J Vis Exp. (135):57676. 10.3791/57676 PMC6101445 29912178

[CIT0066] Smiseth PT, Darwell CT, Moore AJ. 2003. Partial begging: an empirical model for the early evolution of offspring signalling. Proc Biol Sci. 270:1773–1777. 12964978 10.1098/rspb.2003.2444 PMC1691438

[CIT0067] Smiseth PT, Dawson C, Varley E, Moore AJ. 2005. How do caring parents respond to mate loss? Differential response by males and females. Anim Behav. 69:551–559.

[CIT0068] Smiseth PT, Lennox L, Moore AJ. 2007. Interaction between parental care and sibling competition: parents enhance offspring growth and exacerbate sibling competition. Evolution. 61:2331–2339. 17711464 10.1111/j.1558-5646.2007.00192.x

[CIT0069] Smiseth PT, Moore AJ. 2002. Does resource availability affect offspring begging and parental provisioning in a partially begging species? Anim Behav. 63:577–585.

[CIT0070] Smiseth PT, Moore AJ. 2004. Signalling of hunger when offspring forage by both begging and self-feeding. Anim Behav. 67:1083–1088.

[CIT0071] Smiseth PT, Ward RJS, Moore AJ. 2006. Asynchronous hatching in Nicrophorus vespilloides, an insect in which parents provide food for their offspring. Funct Ecol. 20:151–156.

[CIT0072] Steiger S, Gershman SN, Pettinger AM, Eggert A, Sakaluk SK. 2011. Sex differences in immunity and rapid upregulation of immune defence during parental care in the burying beetle, *Nicrophorus orbicollis*. Funct Ecol. 25:1368–1378.

[CIT0073] Stockmaier S, Bolnick DI, Page RA, Carter GG. 2020. Sickness effects on social interactions depend on the type of behaviour and relationship. J Anim Ecol. 89:1387–1394. 32108343 10.1111/1365-2656.13193

[CIT0074] Stroeymeyt N, Grasse AV, Crespi A, Mersch DP, Cremer S, Keller L. 2018. Social network plasticity decreases disease transmission in a eusocial insect. Science. 362:941–945. 30467168 10.1126/science.aat4793

[CIT0075] Therneau T . 2015. A Package for Survival Analysis in S. version 2.38.

[CIT0076] Vale PF, Siva-Jothy JA, Morrill A, Forbes MR. 2018. The influence of parasites. In: Córdoba-Aguilar A, González-Tokman D, González-Santoyo I, editors. Insect behavior: from mechanisms to ecological and evolutionary consequences. Oxford: Oxford University Press. p. 273–291.

[CIT0077] Van Kerckhove K, Hens N, Edmunds WJ, Eames KT. 2013. The impact of illness on social networks: implications for transmission and control of influenza. Am J Epidemiol. 178:1655–1662. 24100954 10.1093/aje/kwt196 PMC3842903

[CIT0078] Venesky MD, Parris MJ, Storfer A. 2009. Impacts of *Batrachochytrium dendrobatidis* infection on tadpole foraging performance. Ecohealth. 6:565–575. 20135192 10.1007/s10393-009-0272-7

[CIT0079] Vilcinskas A, Mukherjee K, Vogel H. 2013. Expansion of the antimicrobial peptide repertoire in the invasive ladybird *Harmonia axyridis*. Proc Biol Sci. 280:20122113. 23173204 10.1098/rspb.2012.2113 PMC3574431

[CIT0080] Vilcinskas A, Stoecker K, Schmidtberg H, Röhrich CR, Vogel H. 2013. Invasive harlequin ladybird carries biological weapons against native competitors. Science. 340:862–863. 23687046 10.1126/science.1234032

[CIT0081] Wang Y, Rozen DE. 2018. Gut microbiota in the burying beetle, *Nicrophorus vespilloides*, provide colonization resistance against larval bacterial pathogens. Ecol Evol. 8:1646–1654. 29435240 10.1002/ece3.3589 PMC5792511

[CIT0082] Williams GC . 1966. Natural selection, the costs of reproduction, and a refinement of Lack’s principle. Am Nat. 100:687–690.

[CIT0083] Ziadie MA, Ebot-Ojong F, McKinney EC, Moore AJ. 2019. Evolution of personal and social immunity in the context of parental care. Am Nat. 193:296–308. 30720366 10.1086/701122

